# Before-and-After Study of the First Four Years of the Enhanced Recovery after Surgery (ERAS^®^) Programme in Older Adults Undergoing Elective Colorectal Cancer Surgery

**DOI:** 10.3390/ijerph192215299

**Published:** 2022-11-19

**Authors:** Cristina Martínez-Escribano, Francisco Arteaga Moreno, David Cuesta Peredo, Francisco Javier Blanco Gonzalez, Juan Maria De la Cámara-de las Heras, Francisco J. Tarazona Santabalbina

**Affiliations:** 1 Anaesthesiology, Resuscitation and Therapeutics of Pain, Hospital Universitario de la Ribera, Ctra de Corbera, km 1, 46600 Alzira, Valencia, Spain; 2 Medical School, Universidad Católica de Valencia San Vicente Mártir, 46001 Valencia, Valencia, Spain; 3 Quality of Care Department, Hospital Universitario de la Ribera, 46600 Alzira, Valencia, Spain; 4 General Surgery Service, Hospital Universitario de la Ribera, 46600 Alzira, Valencia, Spain; 5 Library Service, Hospital Universitario La Ribera, 46600 Alzira, Valencia, Spain; 6 Geriatric Medicine Department, Hospital Universitario de la Ribera, 46600 Alzira, Valencia, Spain; 7 Centro de Investigación Biomédica en Red Fragilidad y Envejecimiento Saludable (CIBERFES), 28029 Madrid, Spain

**Keywords:** colorectal surgery, geriatric assessment, ERAS, postoperative complications, older patients

## Abstract

Background: The aim of this study was to determine whether the inclusion of older patients undergoing elective colorectal cancer resection in the Enhanced Recovery After Surgery (ERAS^®^) programme could improve clinical outcomes during hospital admission. Methods: A before-and-after study in ≥70-year-old patients electively admitted for colorectal cancer resection was designed. In total, 213 patients were included in the ERAS^®^ group, and 158 were included in the control group. Results: The average age was 77.9 years old (SD 5.31) and 57.14% of them were men, with a Charlson Index score of 3.42 (SD 3.32). The ERAS^®^ group presented a lower transfusion rate of 42 (19.7%), compared to 75 (47.5%) in the control group (*p* < 0.001). The crude odds ratio (OR) for transfusion was 0.27 (95% CI 0.17–0.43; *p* < 0.001), and the adjusted odds ratio was 0.26 (95% CI 0.14–0.48; *p* < 0.001). The ERAS^®^ group had a lower percentage of patients with moderate–severe malnutrition on admission, at 23.4% (37 patients) against 36.2% in the control group (42 patients) (*p* = 0.023), with an OR of 0.47 (95% CI 0.29–0.75; *p* < 0.002) and an adjusted OR of 0.48 (95% CI 0.29–0.78; *p* = 0.003). The number of patients who required admission to the intensive care unit (ICU) was also markedly lower: 54 from the ERAS^®^ group (25.4%) versus 71 from the control group (44.9%) (*p* < 0.001). Conclusions: The inclusion of ≥70-year-old adults in the ERAS^®^ programme resulted in a decrease in transfusions, number of erythrocyte concentrates transfused, and number of ICU admissions, along with improved nutritional status.

## 1. Introduction

Cancer is the leading cause of death in adults in developed countries [1]. Globally, colorectal cancer (CRC) is the third most common cancer in men and the second most common in women. In Spain, CRC became the leading cause of death in women in 2020, surpassing breast cancer [2]. Seven out of ten patients diagnosed with CRC [3] and about 60% of patients undergoing elective or urgent surgery due to colorectal cancer are over 65 years of age [4].

Surgery plays a key role in CRC treatment [5] but is associated with a high complication rate that can range from 8% to 63% [6] and global perioperative mortality of between 1% and 12% [7]. Old age adds further mortality and perioperative complications, hampers functional recovery, and increases costs [8]. Although some studies found no differences when age was the only factor taken into consideration [9], ageing-related factors such as frailty and the presence of geriatric syndromes were associated with an increase in mortality and morbidity [10,11]. Similarly, factors such as anaemia and malnutrition, which are common in gastrointestinal pathology, have been associated with worse postoperative outcomes.

Anaemia is an independent risk factor for complications, prolonged hospital stay, and increased mortality in any type of surgery [12,13]. In the case of CRC, it has been related to the advanced stage and proximal location of the tumour, and it has also been attributed prognostic value due to its relationship with overall survival and cancer-specific survival [14]. It is common in older adults [15], especially in the case of colorectal neoplasm, where blood loss is frequent [16]. Moreover, the transfusion rate itself increases complications and mortality [17,18]. Malnutrition is another strong predictor of morbidity, mortality, prolonged hospitalisation, and readmissions [19]. Consequently, the Spanish Multimodal Rehabilitation Group (GERM) and the European Society of Nutrition and Metabolism (ESPEN) emphasise screening for and correcting nutritional deficiencies before surgery [20,21] within enhanced recovery programmes like Fast-Track or ERAS^®^ (Enhanced Recovery After Surgery) [22].

These programmes were developed in order to reduce surgical stress, accelerate recovery, and improve the postoperative outcome in patients undergoing colorectal surgery [23]. The implementation of ERAS^®^ programmes has been shown to reduce complications and shorten hospital stays over the last few years, improving the cost-effectiveness of these processes as a result [24]. These improved results have been evinced for both scheduled and urgent surgeries [25,26]. Several studies have shown the safety of these early recovery programmes [27], but it remains to be seen to what extent these improve hospital outcomes in older adults.

The aim of this study was to assess whether the ERAS^®^ programme implemented in our hospital for ≥70-year-old patients improved their nutritional status and reduced their transfusion rate, postoperative complications, hospital stay, and mortality.

## 2. Materials and Methods

A quasi-experimental before-and-after study in a hospital environment was designed to include all patients consecutively admitted for elective surgery due to colorectal neoplasm to the General Surgery Department of *Hospital Universitario La Ribera* (HULR) from 1 January 2011 to 31 December 2019. The sample consisted of 213 patients who were included in the ERAS^®^ programme since its introduction in 2016 and a further 158 patients previously operated on and treated via traditional means who were included in the control group between 1 January 2011 to 31 December 2015. A bivariate analysis was completed between two periods in the control group (1 January 2011 to 30 June 2013 versus 1 July 2013 to 31 December 2015) to check the homogeneity in the clinical practice during these two periods. No statistical differences were found between groups. HULR is a tertiary care hospital and covers a population of 263,001 inhabitants, of which 19.4% are people aged 65 and over.

### 2.1. Eligibility Criteria

#### 2.1.1. Inclusion Criteria

Patients aged 70 or over electively admitted to the General and Gastrointestinal Surgery Department to undergo curative surgery (which seeks to remove the entire tumour, nearby lymph nodes included) for colorectal cancer resection, stage I–III at diagnosis, were included.

#### 2.1.2. Exclusion Criteria

The study excluded emergency surgery hospital admissions, patients with metastasis at the time of diagnosis, those who had relapsed or were receiving palliative surgery, and patients with an expectation of less than 6 months, according to the Palliative Prognosis Score (PaP Score) [28]. In total, 9.2% of patients from the initial ERAS group and 8.7% from the initial control group did not fulfil the eligibility criteria and were excluded from the sample. The causes were basically progression of the disease at the time of surgery that led to a palliative approach, unresectability, or rapid deterioration of the patient that did not allow adherence to the programme.

### 2.2. Sample Size

The sample size was calculated considering a transfusion rate of 50% prior to the onset of the ERAS^®^ protocols, with an estimated reduction in said rate of 20% by fixing alpha and beta error values of 5%. These data evidenced the need to include a minimum of 154 patients per group. Subsequently, a calculation of the power of the study with the drafted sample was carried out, obtaining a power of 99.5%.

### 2.3. Intervention

The ERAS^®^ protocol developed for the preoperative period in HULR consists of the diagnosis and treatment of anaemia (haemoglobin levels < 13 g/dL in men and <12 g/dL in women) through dosing ferric carboxymaltose, depending on the Hb levels and according to the medication data sheet, 2 to 4 weeks prior to surgery [29]. An assessment of nutritional status through the Controlling Nutritional Status (CONUT) score was performed. Patients classified as risk-free or with a slight risk of malnutrition (CONUT 0–4) were given dietary advice. Those who were classified with moderate–severe risk (CONUT > 4) were prescribed enteral supplements [30]. All patients were referred to a specialised physiotherapist who instructed them in the management of a respiratory incentive to improve lung function and assigned them an exercise chart taking into account several characteristics of the patient. Throughout this time, a telephonic follow-up was carried out by the nursing staff of the surgery unit, who coordinated the process and stayed in contact with patients and their families. One of the fundamental objectives was that the time between diagnosis and surgery should not exceed 4–5 weeks.

From admission to discharge, patients were treated following the recommendations of the 2014 Enhanced Recovery in Abdominal Surgery (RICA) clinical pathway, elaborated by the Spanish Multimodal Rehabilitation Group (GERM) [31].

Patients assisted before the introduction of the ERAS^®^ protocol in our hospital were treated as recommended via the clinical pathways of both anaesthesia and general surgery of that time, which involved no nutritional intervention, nor iron administration, nor physical activity guides.

### 2.4. Variables and Outcomes

Several variables were included: demographic variables (age and sex), anthropometric variables (weight, height, and body mass index (BMI)), frailty according to the Balducci Scale (validated for geriatric oncology because of its simplicity and agility) [32] and presence of geriatric syndromes, comorbidity and Charlson Index [33], tumour location and staging; laboratory data, proinflammatory state markers (C-reactive protein (PCR) and procalcitonin) and hospital process data, complications, intensive care unit admissions, hospital stay, number of reinterventions, readmissions, hospital mortality, and 1-year mortality.

The principal outcome was to determine whether the introduction of the ERAS^®^ programme reduced the anaemia and malnutrition incidence at the time of surgery. It was considered that a patient suffered from anaemia if their haemoglobin levels were <13 g/dL in men and <12 g/dL in women, according to the WHO (World Health Organisation) classification [34]. The transfusion incidence (percentage of transfused patients out of the total number of operated patients) and the Total Transfusion Index (number of erythrocyte concentrates used per operated patient) were registered. The nutritional status was assessed at admission and discharge by scoring on the CONUT nutritional index, calculated from albumin serum levels, overall cholesterol, and lymphocyte count. Depending on the calculated value, patients were classified as risk-free or with slight risk of malnutrition if the total was CONUT ≤ 4 and as moderate–severe risk if CONUT > 4.

The effect of our intervention was also studied to determine whether it was reflected in the incidence of complications, both medical (delirium, heart failure or respiratory insufficiency, infections, etc.) and surgical (suture dehiscence, intestinal pseudo-obstruction (understood as a lack of gastrointestinal transit and oral tolerance set out 5 days post-surgery) and surgical wound infection), in the number of ICU admissions and reinterventions, hospital stay, readmissions rate, hospital mortality, and 1-year mortality.

### 2.5. Statistical Analysis

Data were analysed using version 22 of the statistical software program SPSS (SPSS Inc., Chicago, IL, USA).

A description of the qualitative variables (including dichotomous variables) through the use of absolute and relative frequencies was made. For quantitative variables, measures of central tendency (mean) were used, along with measures of dispersion (standard deviation (SD) or interquartile range (IQR)), depending on whether or not variables met normal distribution criteria as determined using the Kolmogorov–Smirnov test.

A bivariate calculation was performed for the variables mentioned in the main and secondary objectives. Student’s *t*-test was used for quantitative variables with a normal distribution, and the Chi-Square test was used for qualitative variables. A binary logistic regression was made for the “transfused erythrocyte concentrates” variable by calculating the crude and adjusted odds ratio (OR) for the following variables: age, sex, Charlson Index, frailty, tumour stage, and CONUT score at hospital admission.

A multiple logistic regression model was built in order to study the need for transfusions, presence of malnutrition, need for ICU admissions, and hospital stays of ≤6 days. Survival was estimated using the Kaplan–Meier statistical method, and the difference between groups was estimated using the Mantel–Haenszel test.

Variables related to 1-year mortality were assessed using Cox’s proportional hazards model and defined as deaths that occurred in the following 365 days.

Moreover, the Number Needed to Treat (NNT) calculation was performed using the NNT macro for SPSS [34], in order to determine the need for transfusion and the presence of malnutrition, ICU admissions, and stays longer than 6 days.

The significance threshold was set to a value of *p* < 0.05.

## 3. Results

In total, 371 patients were included in the study period (213 in the ERAS^®^ group and 158 in the control group), of whom 212 (57.1%) were men. The average age was 77.9 years old (ranging from 70 to 96 years old), and the average Charlson Index was 3.4 (SD 3.3). The most frequent tumour location was the colon, with an incidence of 55%. No differences were found in relation to sex between groups in the bivariate analysis, but age differences were found: the average age was significantly higher in the ERAS^®^ group, 78.5 years old (SD 5.14) compared to 77.0 (SD 5.41) in the control group (*p* = 0.009, Table 1). Likewise, the ERAS^®^ group presented a higher incidence of myocardial ischaemia, heart failure, diabetes mellitus, and frailty, together with a significantly higher localisation rate of colon neoplasm—61.7% compared to 55.1% in the control group (*p* = 0.018). Table 1 presents the main features of each group at hospital admission.

The percentage of laparoscopies was substantially higher in the ERAS^®^ group—66% compared to 45% in the control group (*p* < 0.001). The duration of surgery was also higher, with 197 min (SD 65.32) in the ERAS^®^ group against 170 min (SD 64.0) in the control group (*p* < 0.001). The number of reoperated patients was higher in the ERAS^®^ group, at 10.3% compared to 4.4% in the control group (*p* = 0.049, Table 2).

There were no differences in haemoglobin levels at admission or at discharge, but differences in the minimum levels of haemoglobin registered during the hospital stay were significant. A meaningful reduction in the transfusion rate was observed in the ERAS^®^ group—19% against 47% in the control group (*p* < 0.001). The Total Transfusion Index was considerably lower in the ERAS^®^ group, at 0.52 (SD 1.24) compared to 1.68 (SD 2.75) in the control group (*p* < 0.001, Table 2). There were no differences in the overall incidence of medical or surgical complications (Table 2).

It was observed in the multivariate analysis that patients included in the ERAS^®^ group presented a crude OR of transfusion of 0.27 (95% confidence interval (95% CI) 0.17–0.43; *p* < 0.001), with an adjusted OR of 0.26 (CI 95% 0.14–0.48; *p* < 0.001) (Table 3). The Number Needed to Treat (NNT) in the ERAS^®^ programme in order to avoid transfusion was 4 (Table 4).

In regard to nutritional assessment, the ERAS^®^ group presented a statistically lower percentage of moderate or severe malnutrition (estimated using the CONUT score) at admission—23.6% against 40.1% in the control group (*p* = 0.010). In fact, the average score in the CONUT nutritional screening was also significantly lower upon admission in ERAS^®^ group patients—2.7 (SD 2.8) against 3.64 (SD 3.6) in the control group (*p* = 0.012). The improvement in nutritional status was also reproduced at hospital discharge, at which point patients in the ERAS^®^ group had a CONUT score of 4.69 (SD 2.40) compared to 5.72 (SD 2.86) in the control group (*p* = 0.003).

It was observed in the multivariate analysis that patients in the ERAS^®^ group presented a lower risk of moderate or severe malnutrition at admission, with an OR of 0.47 (95% CI 0.29–0.75; *p* < 0.002) and an adjusted OR of 0.48 (95% CI 0.29–0.78; *p* = 0.003). The OR of presenting moderate–severe malnutrition at discharge was significantly lower in the ERAS^®^ programme patients, at 0.52 (95% CI 0.34–0.80; *p* < 0.003), with an adjusted OR of 0.55 (95% CI 0.36–0.85; *p* = 0.007) (Table 3). The NNT calculated in a patient in order to avoid malnutrition at admission was 7 (Table 4).

This lower transfusion rate and improved nutritional status translated into a higher percentage of patients with a hospital stay of ≤6 days in the ERAS^®^ group—26.8% against 17.7% of the control group (*p* = 0.046). There were no differences in the total hospital stay, the mean being 11.5 days (SD 10.2) in the ERAS^®^ group compared to 11.4 days (SD 8.58) in the control group (*p* = 0.926). The increased complexity of the profile of patients in the ERAS^®^ group did not lead to a longer stay, and the percentage of patients that required admission to the ICU was significantly lower—25.4% compared to 44.9% in the control group (*p* ≤ 0.001). No differences were found in the overall incidence of surgical or medical complications (Table 3). Further, no differences in hospital mortality or 1-year mortality were found.

## 4. Discussion

The data from our study show that the participation of older patients in the ERAS^®^ programme since its implementation in 2016 has resulted in a substantial decrease in the following variables: the number of patients requiring transfusion, number of erythrocyte concentrates used, number of patients with moderate or severe malnutrition at the moment of surgery and at discharge, and ICU admissions.

The progressive population dynamics are leading to an increase in older-age patients that will eventually need surgical procedures [34,35], and this can be seen in the higher age of the intervention group compared to the control group. The reduction in the number of patients requiring transfusion observed in this study after the implementation of the enhanced recovery programme was previously also described for patients undergoing thoracic and orthopaedic surgery [36,37].

In this study, the probability of receiving a transfusion was related to the laparoscopic approach and initial haemoglobin, but the probability of requiring transfusion decreased for all patients who participated in the ERAS^®^ programme, regardless of preoperative anaemia levels.

This outcome can be related to the intravenous administration of ferric carboxymaltose before surgery [29,38], the application of more restrictive transfusion strategies, and less invasive surgical techniques. These proceedings are included in the ERAS^®^ protocol [39,40].

Although nutritional deficiency is frequent in patients with neoplasm, especially in gastrointestinal neoplasm, the protocol managed to significantly reduce the percentage of patients with moderate or severe malnutrition at admission, thanks to the presurgical nutritional intervention that was carried out. Moreover, the risk of malnutrition is high in hospitalised patients and increases as hospital stays are prolonged [41]. In our case, the number of patients with malnutrition increased with the stay duration, but it was significantly lower in the ERAS^®^ group than in the control group.

These results show the need for pre-surgical prehabilitation protocols like ERAS^®^, through which patients who undergo elective colorectal surgery can be optimised and have an early recovery. In this regard, the diagnosis and early control of anaemia and malnutrition are crucial for decreasing anaemia incidence at the moment of surgery, reducing the intake of blood-derivative drugs [36,39,42,43], and improving nutritional status [19,21,40,44].

No reduction was observed in the incidence of medical and surgical complications, hospital stay, or mortality, as reported in previous studies [38]. However, it is worth noting that patients in the ERAS^®^ group presented higher prevalence of frailty, diabetes, and chronic heart disease, which may have influenced the absence of significant differences. In fact, the higher prevalence of frailty in the ERAS^®^ group, as previously described, could be associated with an increase in complications, hospital stay, and readmissions and a reduction in survival [45]; these results correlate better with the presence of frailty than with age, morbidity, or even the severity of the surgical process [10,46]. Despite this more complex profile, the ERAS^®^ group presented a significantly higher proportion of patients with a hospital stay of less than 6 days and a decrease in ICU admissions.

Patients in the ERAS^®^ group underwent laparoscopy to a larger extent than the control group and presented a longer surgery duration, which was not associated with an increment in the incidence of intestinal pseudo-obstruction in the postoperative period. In line with previous studies, our intestinal pseudo-obstruction incidence was high (23.5%), despite the decreased transfusion rate, use of morphics, and guided management of fluid therapy, as all these interventions are included in ERAS^®^ patient management and are also related to this complication [47].

### Limitations

The study presents the typical biases of quasi-experimental studies, such as those of selection, especially the use of a historical cohort as a comparison group, and of confusion [48]. The main limitation of the study results from its nonrandomised design, which led to differences between groups in terms of the prevalence of frailty, diabetes, heart failure, and myocardial ischaemia. The bivariate analysis shows a more complex profile of patients in the intervention group than in the control, which confers greater internal validity to the results obtained by the intervention. A registration of patient adherence to the programme, which would have sustained the obtained outcomes, was not performed. Personal histories from the non-ERAS^®^ group were retrospectively collected, with biases resulting from the loss of information this entails.

## 5. Conclusions

The ERAS^®^ intervention reduced the need for transfusions and the number of transfused erythrocyte concentrates; furthermore, it improved the nutritional status of patients at admission and discharge, reduced ICU admissions, and increased the percentage of patients with hospital stays of less than 6 days. These results suggest that elderly patients can also benefit from participation. Further studies in patients in this age range are necessary to more exactly determine the true potential of the ERAS^®^ programme in these patients.

## Figures and Tables

**Table 1:** Baseline characteristics of the ERAS and control group.

	ERAS (*n* = 213)	Non-ERAS (*n* = 158)	*p*
Age (years), mean (SD)	78.5 (5.14)	77.0 (5.41)	0.009
Sex *n* (%)			
Male	119 (55.9%)	93 (58.9%)	0.597
Female	94 (44.1%)	65 (41.1%)	
BMI, mean (SD)	28.7 (4.49)	28.1 (4.64)	0.681
Charlson Comorbidity Index, mean (SD)	3.37 (3.19)	3.50 (3.53)	0.168
Pathological history			
Frailty signs *n* (%)			
0	72 (33.8%)	73 (46.2%)	0.018
1 or more	141 (66.2%)	85 (53.8%)	
Dementia *n* (%)	5 (2.3%)	5 (3.2%)	0.749
Stroke *n* (%)	18 (8.5%)	12 (7.6%)	0.849
Heart failure *n* (%)	56 (26.3%)	19 (12.0%)	0.001
Myocardial ischaemia *n* (%)	36 (16.9%)	9 (5.7%)	0.001
Chronic pulmonary disease *n* (%)	35 (16.4%)	26 (16.5%)	1.000
Diabetes mellitus *n* (%)	70 (32.9%)	29 (18.4%)	0.002
Chronic renal insufficiency *n* (%)	14 (6.6%)	13 (8.2%)	0.551
ASA score *n* (%)			
I	85 (39.9%)	63 (39.9%)	0.912
II	121 (56.8%)	91 (57.6%)	
III	7 (3.3%)	4 (2.5%)	
Endovenous iron treatment before surgery	114 (53.5%)	0(0%)	<0.001
Tumour location *n* (%)			
Colon	143 (67.1%)	87 (55.1%)	0.018
Rectosigmoid	70 (32.9%)	71 (44.9%)	
Stage *n* (%)			
I	68 (31.9%)	36 (22.8%)	0.014
II	64 (30.0%)	55 (34.8%)	
III	69 (32.4%)	45 (28.5%)	
IV	12 (5.6%)	22 (13.9%)	

Legend: ERAS: Enhanced Recovery After Surgery; BMI: body mass index; ASA: American Society of Anaesthesiologists.

**Table 2:** Results of bivariate analysis of variables during hospital stay and hospital discharge.

	ERAS (*n* = 213)	Non-ERAS (*n* = 158)	*p*
Type of surgery *n* (%)			
Open	73 (34.3%)	76 (49.0%)	<0.001
Laparoscopy	140 (65.7%)	72 (46.5%)	
Surgery duration (minutes), mean (SD)	197 (65.32)	170 (63.99)	<0.001
Haemoglobin (g/dL) at admission, mean (SD)	12.3 (1.70)	12.2 (2.01)	0.492
Anaemia *n* (%)	114 (53.5%)	87 (55.1%)	0.833
Lymphocytes (1.10⁹/L), mean (SD)	2.35 (0.97)	2.41 (1.00)	0.563
Cholesterol (mg/dL), mean (SD)	147.4 (43.09)	134.7 (43.71)	0.006
Albumin (g/dL), mean (SD)	3.64 (0.65)	3.38 (0.80)	0.001
C-reactive protein (mg/mL), mean (SD)	63.4 (68.92)	51.2 (63.90)	0.127
Procalcitonin (mg/mL), mean (SD)	0.46 (1.30)	0.37 (0.85)	0.409
CONUT score at admission, mean (SD)	2.70 (2.79)	3.64 (3.34)	0.009
CONUT > 4 at admission *n* (%)	50 (23.6%)	59 (40.2%)	0.010
Hospital stay (days), mean (DS)	11.5 (10.20)	11.4 (8.58)	0.926
Admissions of at most 6 days *n* (%)	57 (26.8%)	28 (17.7%)	0.046
ICU admissions *n* (%)	54 (25.4%)	71 (44.9%)	<0.001
Adverse events in the postoperative period *n* (%)	124 (53.7%)	103 (65.2%)	0.530
Medical complications *n* (%)	45 (21.1%)	29 (18.4%)	0.599
Delirium *n* (%)	15 (7.0%)	12 (7.6%)	0.843
Heart complications *n* (%)	19 (8.9%)	7 (4.4%)	0.104
Respiratory complications *n* (%)	12 (5.6%)	11 (7.0%)	0.666
Digestive complications *n* (%)	5 (2.3%)	0 (0.0%)	0.075
Urinary tract infection *n* (%)	8 (3.8%)	4 (2.5%)	0.568
Surgical infection *n* (%)	12 (5.6%)	5 (3.2%)	0.321
Surgical complications *n* (%)	57 (26.8%)	53 (33.5%)	0.169
Intestinal pseudo-obstruction *n* (%)	50 (23.5%)	46 (29.1%)	0.232
Suture dehiscence *n* (%)	9 (4.2%)	5 (3.2%)	0.784
Reintervention *n* (%)	22 (10.3%)	7 (4.4%)	0.049
Transfusion rate *n* (%)	42 (19.7%)	75 (47.5%)	<0.001
TTI (SD)	0.52 (1.24)	1.68 (2.75)	<0.001
Minimum haemoglobin (g/dL) at episode, mean (SD)	10.11 (1.38)	9.73 (1.53)	0.013
Haemoglobin at discharge, mean (SD)	11.03 (1.39)	10.76 (1.27)	0.056
Cholesterol at discharge (mg/dL), mean (SD)	132.2 (29.84)	124.6 (33.14)	0.023
Albumin at discharge (g/dL), mean (SD)	3.18 (0.39)	3.04 (0.40)	0.001
CONUT at discharge, mean (SD)	4.69 (2.40)	5.72 (2.86)	0.002
CONUT > 4 at discharge *n* (%)	83 (39.2%)	86 (54.8%)	0.002
Readmissions *n* (%)	5 (2.3%)	4 (2.5%)	1.000
Hospital mortality *n* (%)	9 (4.2%)	4 (2.5%)	0.570
1-year mortality *n* (%)	24 (11.3%)	12 (7.6%)	0.288

Legend: ERAS: Enhanced Recovery After Surgery; TTI: Total Transfusion Index; CONUT: Controlling Nutritional Status; *n*: total number; %: percentage; SD: standard deviation; g: grams; dL: decilitres; mg: milligrams; L: litres.

**Table 3:** Multivariate logistic regression analysis of significant variables in association with participation in the ERAS^®^ programme, including the following adjusted variables: age, sex, Charlson Index, frailty, tumour stage, and CONUT score at admission.

	Crude OR	95% CI	*p*	Adjusted OR	95% CI	*p*
Transfusion	0.27	0.17–0.43	<0.001	0.26	0.14–0.48	<0.001
CONUT > 4 at admission	0.46	0.29–0.76	0.002	0.48	0.29–0.78	0.003
CONUT > 4 at discharge	0.52	0.34–0.80	0.003	0.55	0.36–0.85	0.007
ICU admissions	0.34	0.21–0.57	<0.001	0.42	0.27–0.65	<0.001
LOS ≥ 6 days	1.41	0.73–2.75	0.309	1.39	0.75–2.68	0.311

Legend: CONUT: Controlling Nutritional Status; ICU: intensive care unit; LOS: length of stay; OR: odds ratio; CI: confidence interval.

**Table 4:** Number Needed to Treat in order to benefit from participation in the programme.

	NNT	95% CI
Transfusion	3.6	2.7–5.5
CONUT < 4 at admission	6.9	4.2–18.3
CONUT > 4 at discharge	6.4	3.9–18.7
ICU admissions	5.1	3.4–10.2
LOS ≤ 6 days	4.4	3.2–7.3

Legend: NNT: Number Needed to Treat; CONUT: Controlling Nutritional Status; ICU: intensive care unit; LOS: length of stay; CI: confidence interval.

## Data Availability

Not applicable.
